# Supplementary material for: Intron-encoded cistronic transcripts for minimally invasive monitoring of coding and non-coding RNAs
Source: Nat Cell Biol. 2022 Nov 7;24(11):1666–76. doi: 10.1038/s41556-022-00998-6 (PMC9643161; doi:10.1038/s41556-022-00998-6)
Supplement: Supplementary file 1 — Supplementary Figs. 1–3. [file 41556_2022_998_MOESM1_ESM.pdf]

nature cell biology

Technical Report https://doi.org/10.1038/s41556-022-00998-6

Intron-encoded cistronic transcripts for minimally invasive monitoring of coding and non-coding RNAs

In the format provided by the authors and unedited

Supplementary Information

Intron-encoded cistronic transcripts for minimally-invasive monitoring of coding and non-coding RNAs

Dong-Jiunn Jeffery Truong[1,2]*, Niklas Armbrust[1,2]*, Eva-Maria Lederer[1,2], Julian Geilenkeuser[1,2], Tobias Heinrich Santl[1,2], Maren Beyer[1,2], Sebastian Ittermann[3], Emily Steinmaßl[1,2], Mariya Dyka[1,2], Gerald Raffl[1,2], Teeradon Phlairaharn[1,2], Tobias Greisle[3], Milica Živanić[1,2], Markus Grosch[3], Micha Drukker[3], Gil Gregor Westmeyer[1,2,✉]

[1]Institute for Synthetic Biomedicine, Helmholtz Zentrum München, Neuherberg, Germany

[2]Department of Chemistry & TUM School of Medicine, Technical University of Munich, Munich, Germany

[3]Institute of Stem Cell Research, Helmholtz Zentrum München, Neuherberg, Germany

*both authors contributed equally

[✉]Correspondence: gil.westmeyer@tum.de

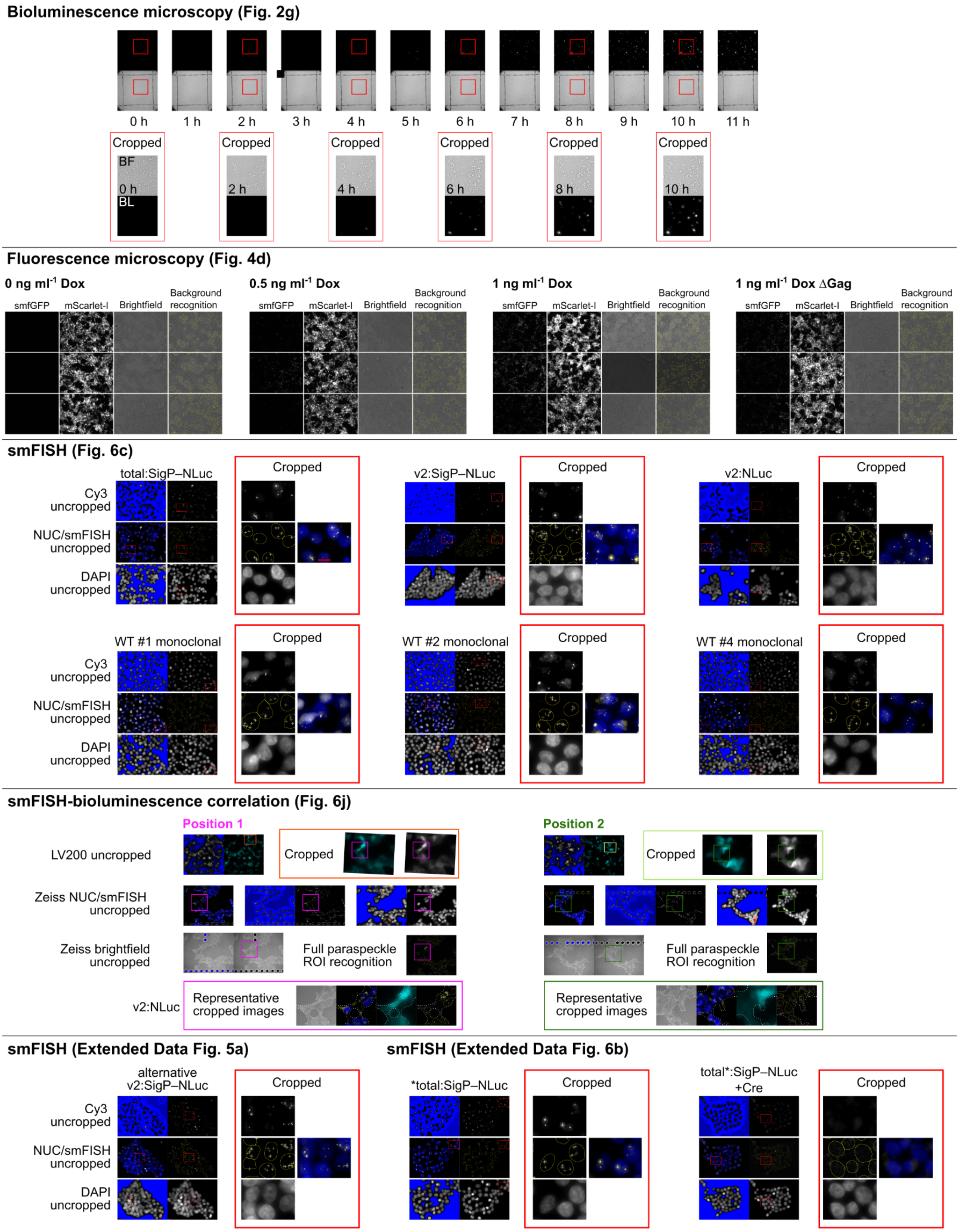

Supplementary Fig. 1 | Uncropped microscopy images.

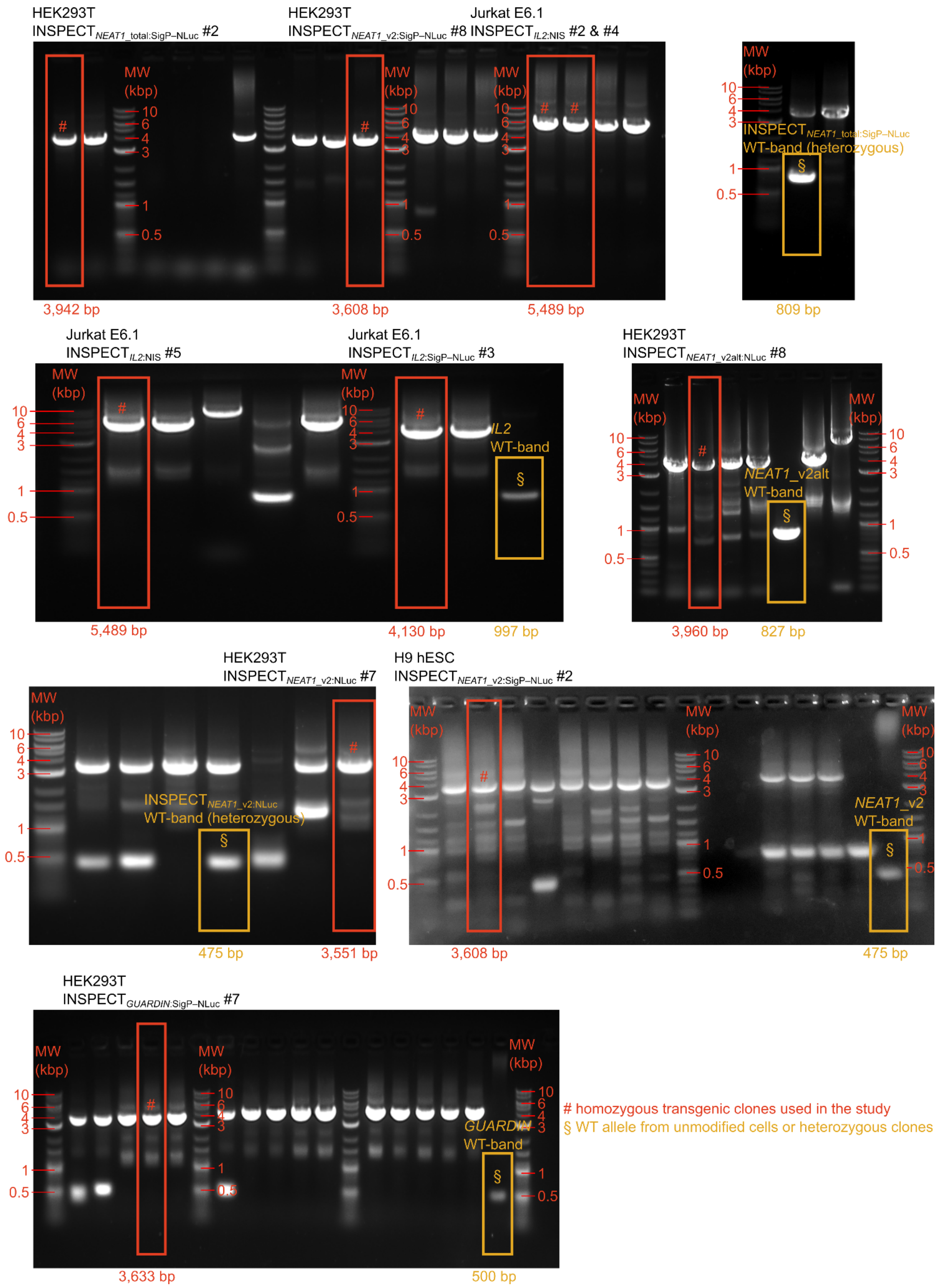

Supplementary Fig. 2 | Genotyping.

FACS batch analysis

Sample 3-1–3-3: INSPECT
Sample 4-1–4-2: INSPECT 5'-xrRNA 3'-XAP1
Sample 5-1–5-3: INSPECT 5'-xrRNA 3'-HCV-UTR

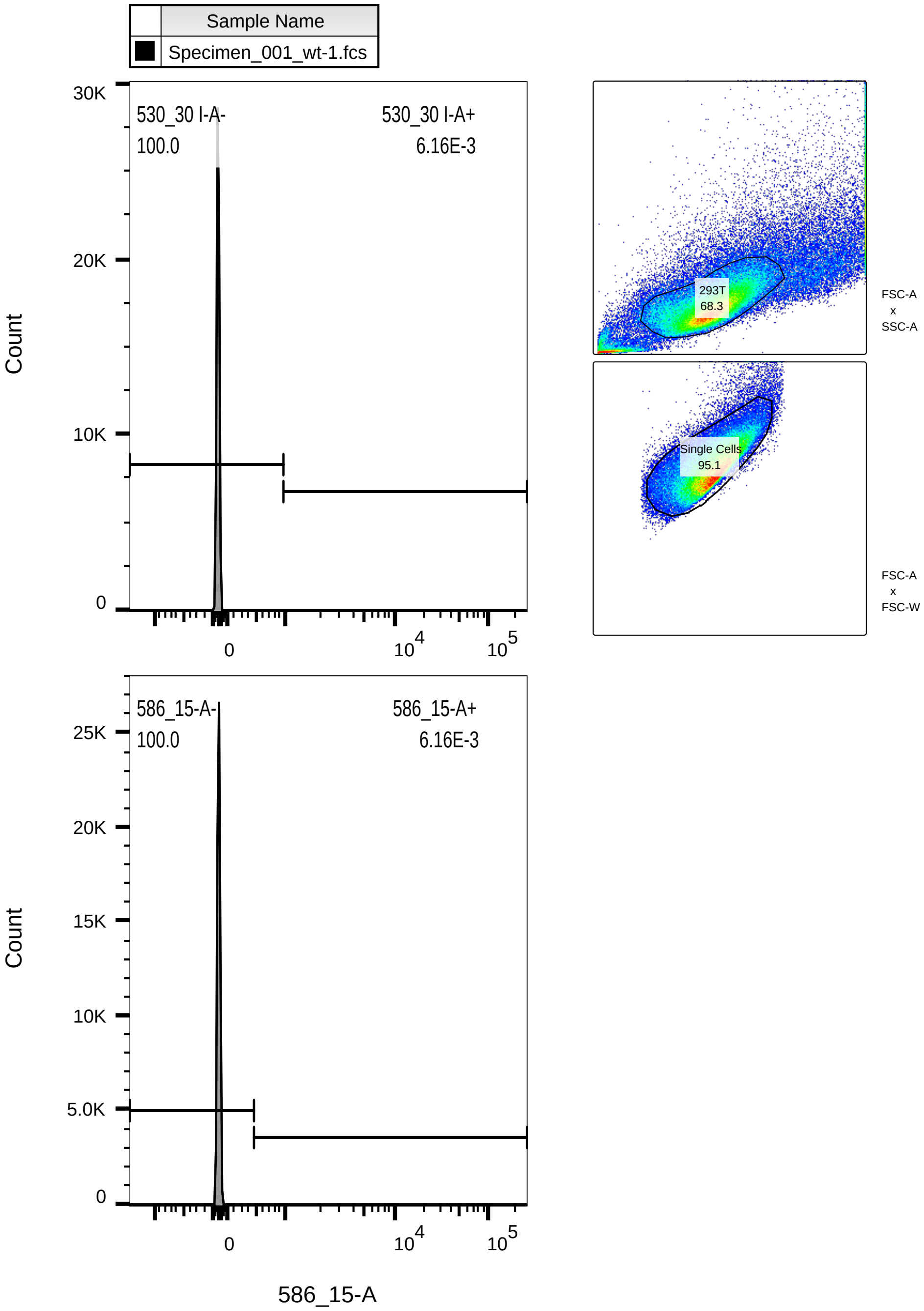

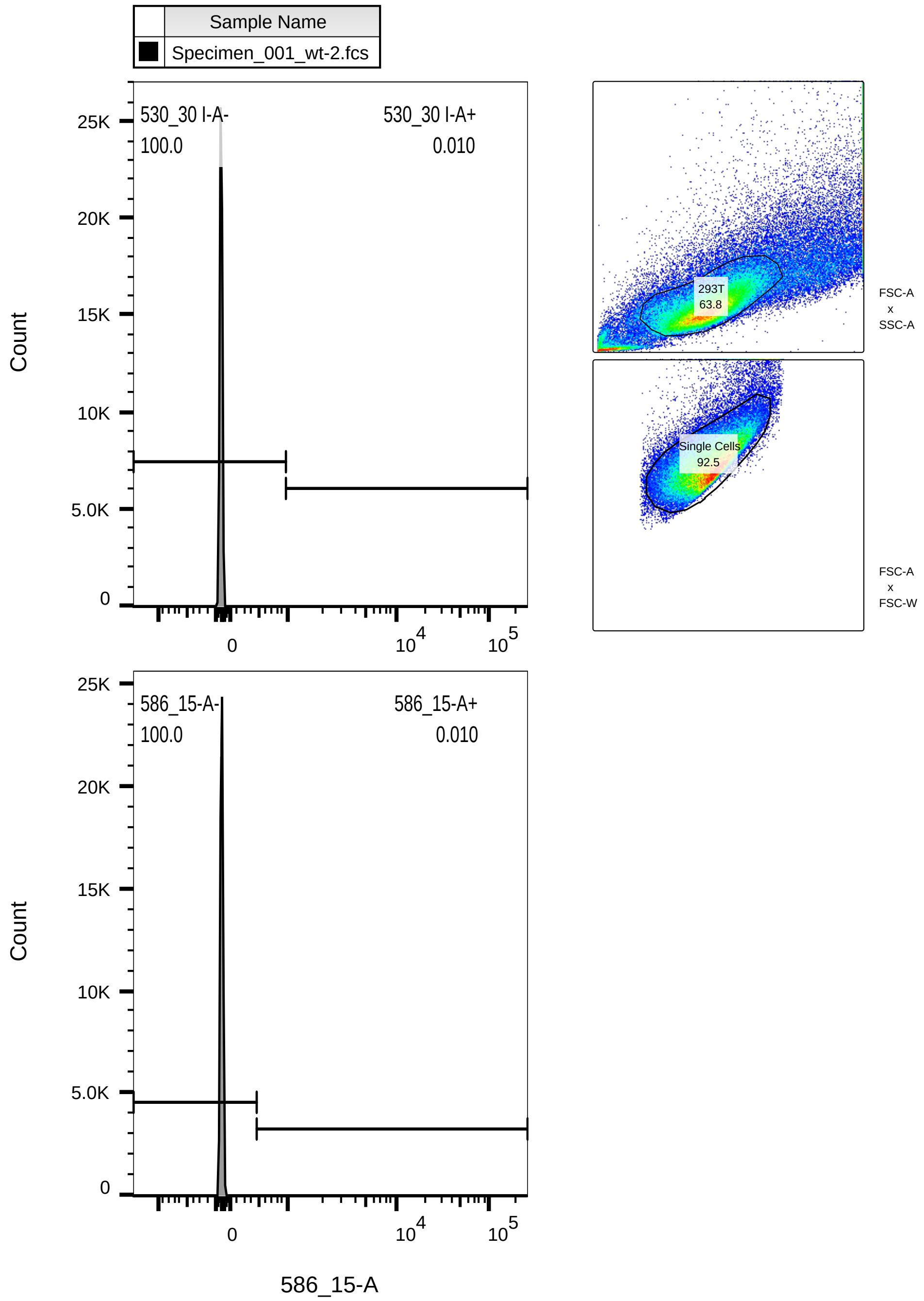
Sample Name
Specimen_001_wt-2.fcs
530_30 I-A-
100.0
530_30 I-A+
0.010
Count
25K
20K
15K
10K
5.0K
0
0
10^4
10^5
293T
63.8
FSC-A
x
SSC-A
Single Cells
92.5
FSC-A
x
FSC-W
586_15-A-
100.0
586_15-A+
0.010
586_15-A

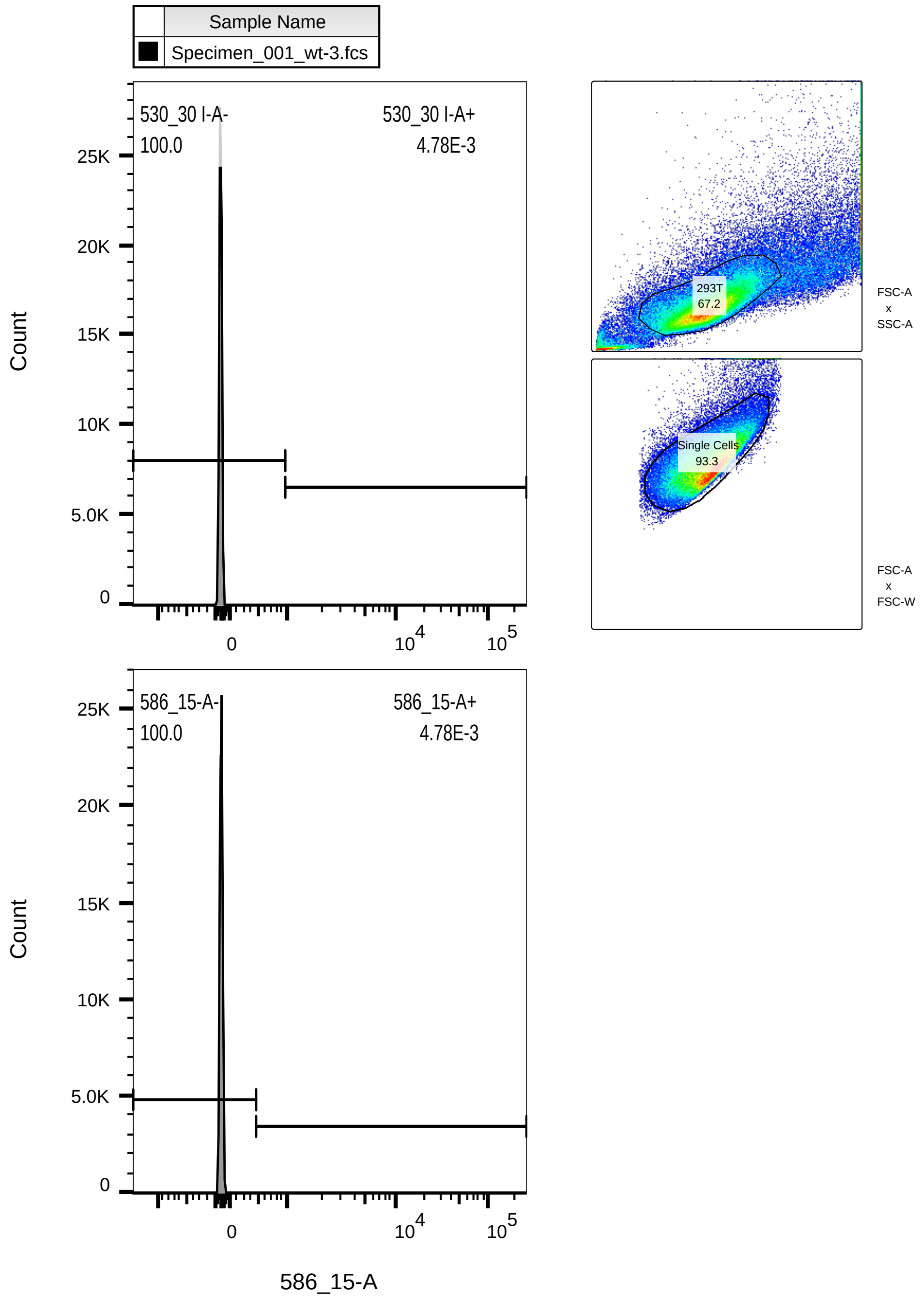
Sample Name
Specimen_001_wt-3.fcs
530_30 I-A-
100.0
530_30 I-A+
4.78E-3
Count
25K
20K
15K
10K
5.0K
0
0
10^4
10^5
293T
67.2
FSC-A
x
SSC-A
Single Cells
93.3
FSC-A
x
FSC-W
586_15-A-
100.0
586_15-A+
4.78E-3
Count
25K
20K
15K
10K
5.0K
0
0
10^4
10^5
586_15-A

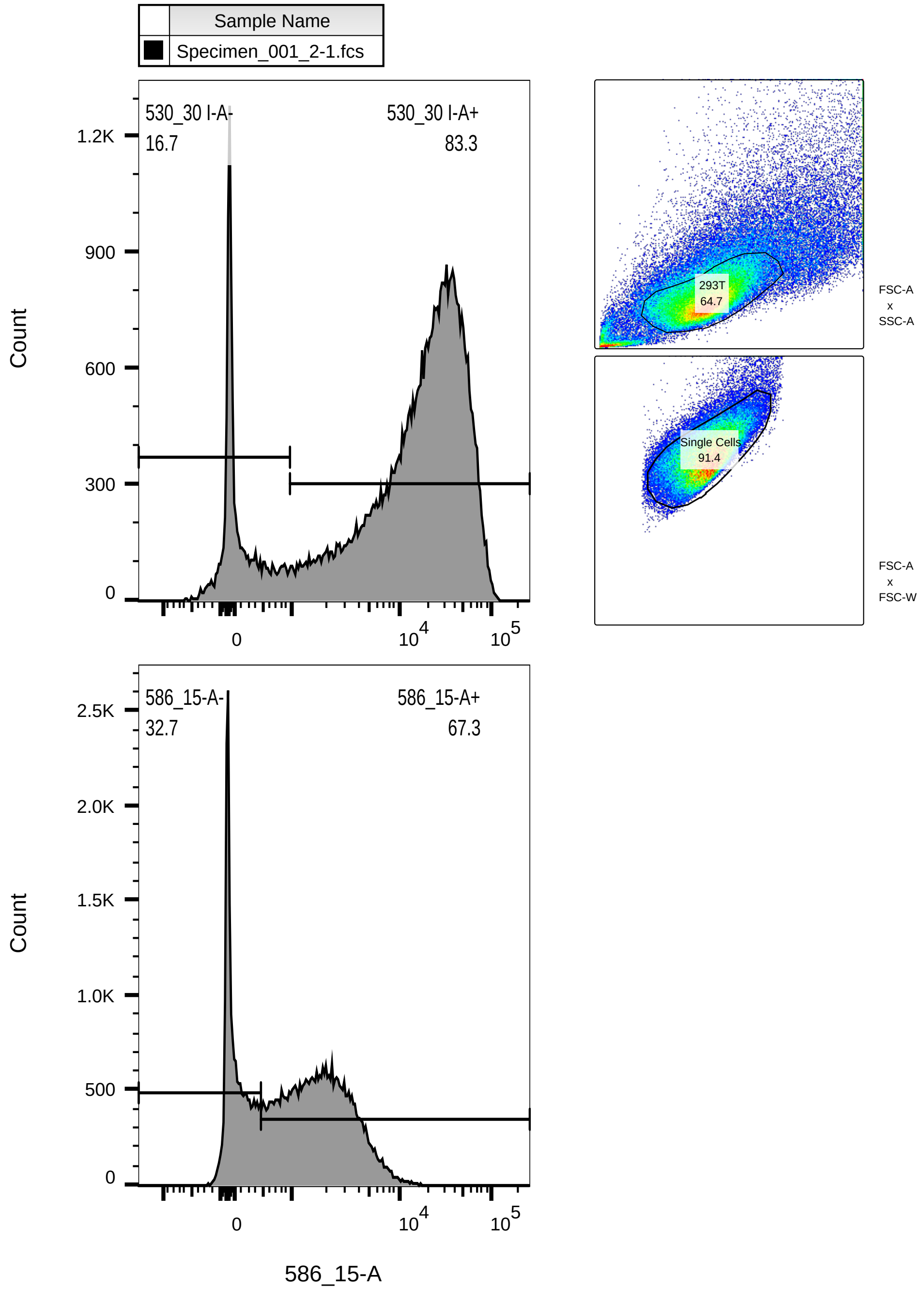
Sample Name
Specimen_001_2-1.fcs
530_30 I-A-
16.7
530_30 I-A+
83.3
Count
1.2K
900
600
300
0
10^4
10^5
293T
64.7
FSC-A
x
SSC-A
Single Cells
91.4
FSC-A
x
FSC-W
586_15-A-
32.7
586_15-A+
67.3
2.5K
2.0K
1.5K
1.0K
500
586_15-A

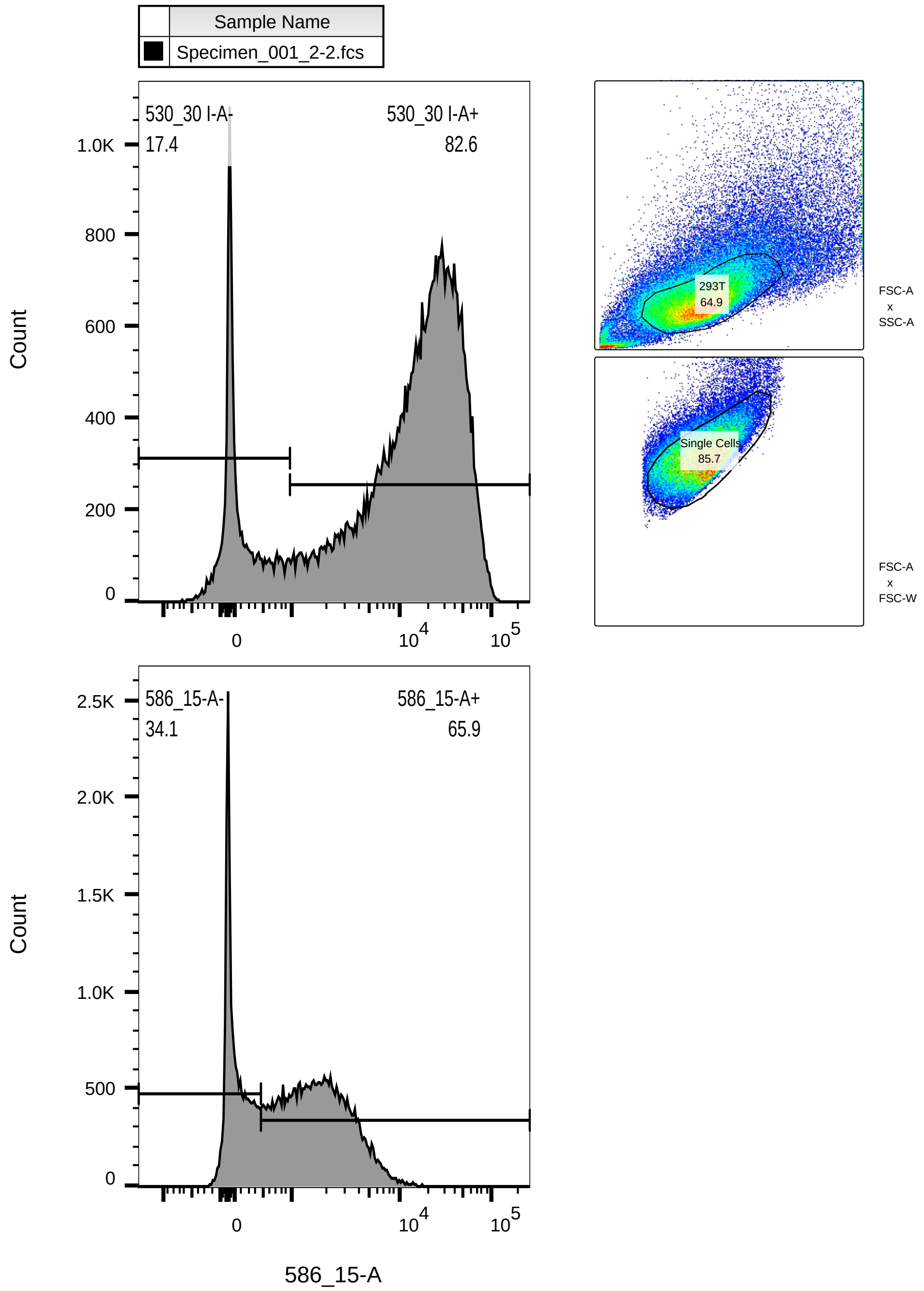

Sample Name
Specimen_001_2-2.fcs
530_30 I-A-
17.4
530_30 I-A+
82.6
Count
1.0K
800
600
400
200
0
10^4
10^5
293T
64.9
FSC-A x SSC-A
Single Cells
85.7
FSC-A x FSC-W
586_15-A-
34.1
586_15-A+
65.9
2.5K
2.0K
1.5K
500
586_15-A

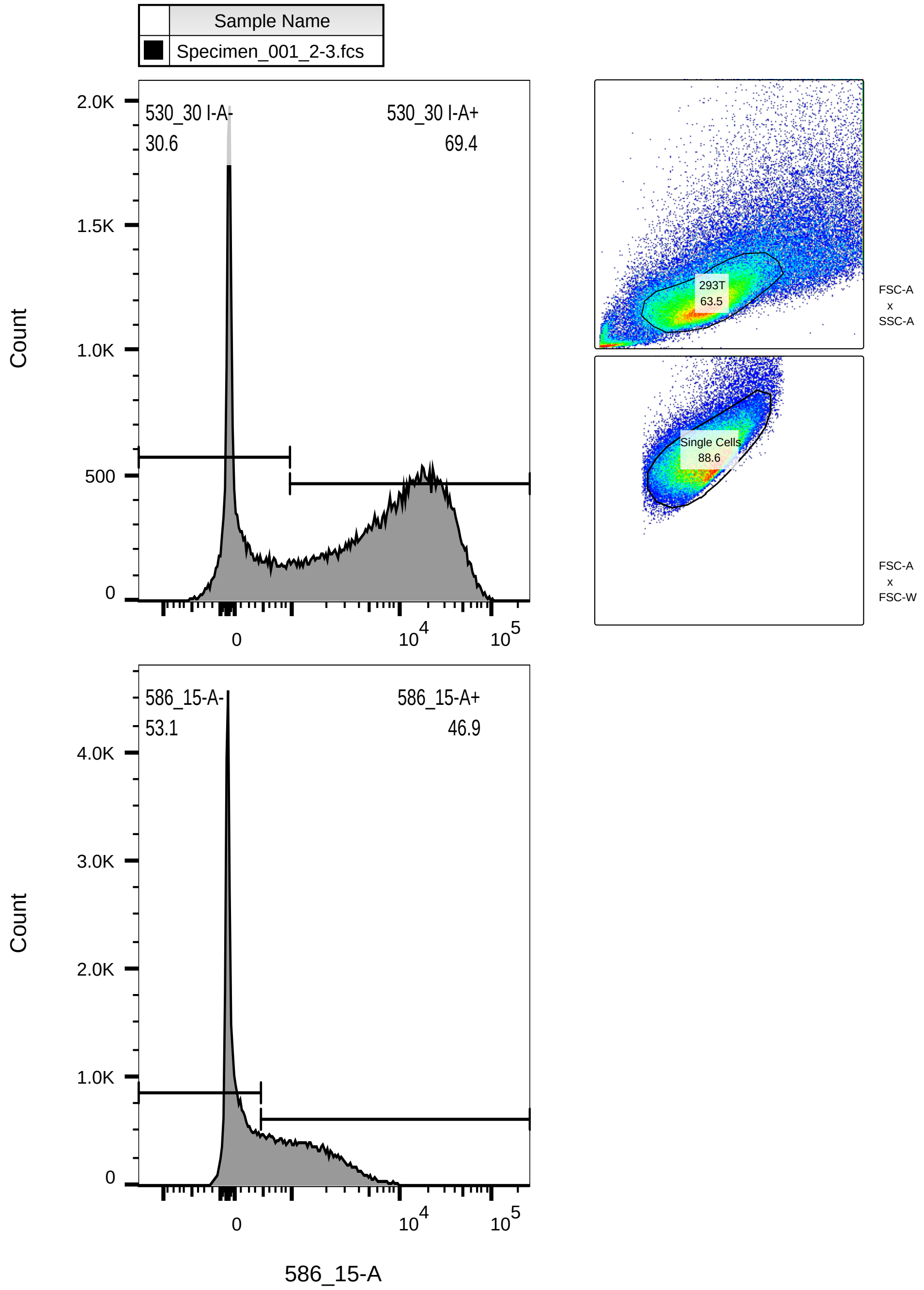

Sample Name
Specimen_001_2-3.fcs
530_30 I-A-
30.6
530_30 I-A+
69.4
Count
586_15-A-
53.1
586_15-A+
46.9
586_15-A
293T
63.5
FSC-A x SSC-A
Single Cells
88.6
FSC-A x FSC-W

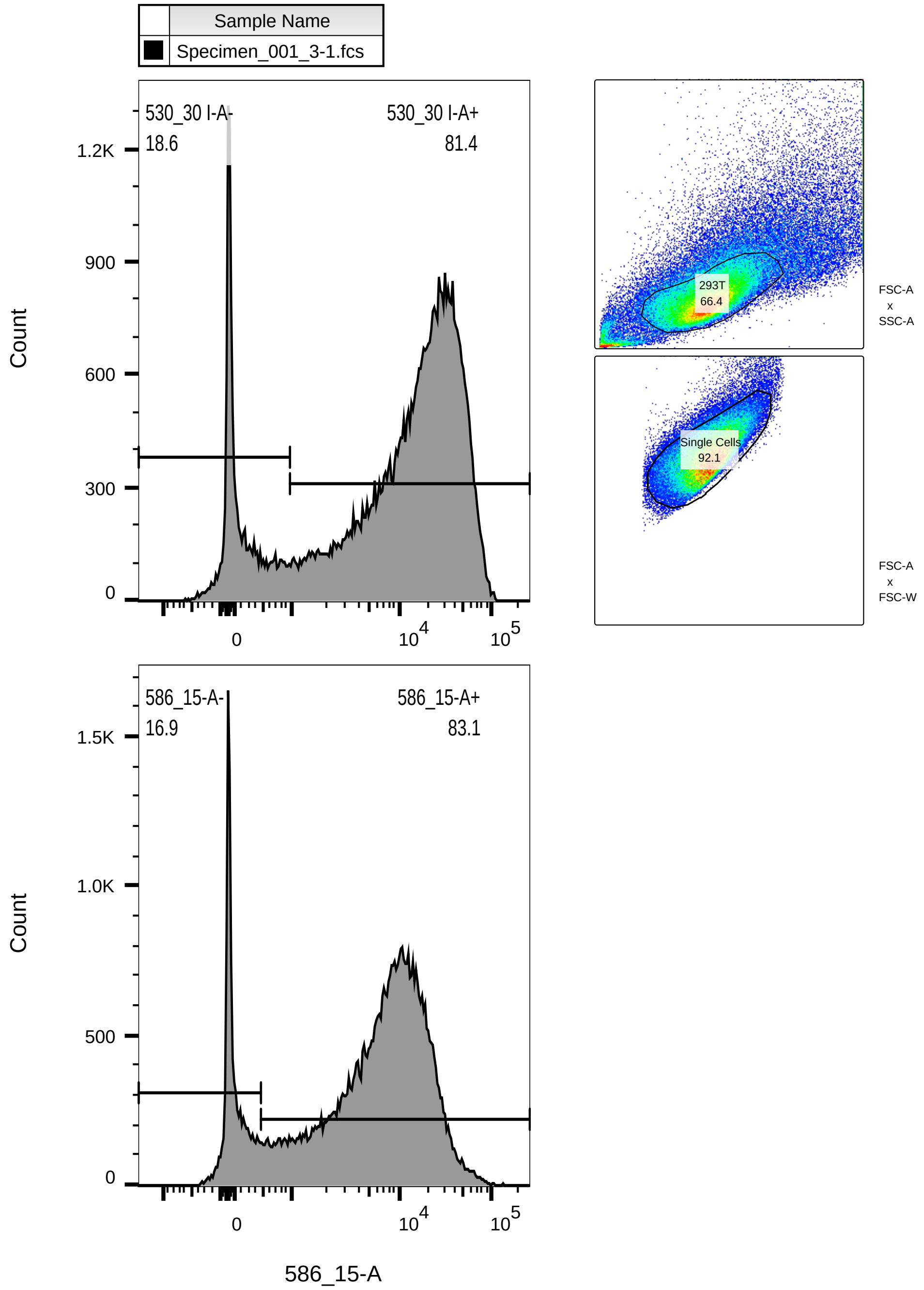
Sample Name
Specimen_001_3-1.fcs
530_30 I-A-
18.6
530_30 I-A+
81.4
1.2K
900
600
300
0
Count
0
10^4
10^5
586_15-A-
16.9
586_15-A+
83.1
1.5K
1.0K
500
0
Count
0
10^4
10^5
586_15-A
293T
66.4
FSC-A
x
SSC-A
Single Cells
92.1
FSC-A
x
FSC-W

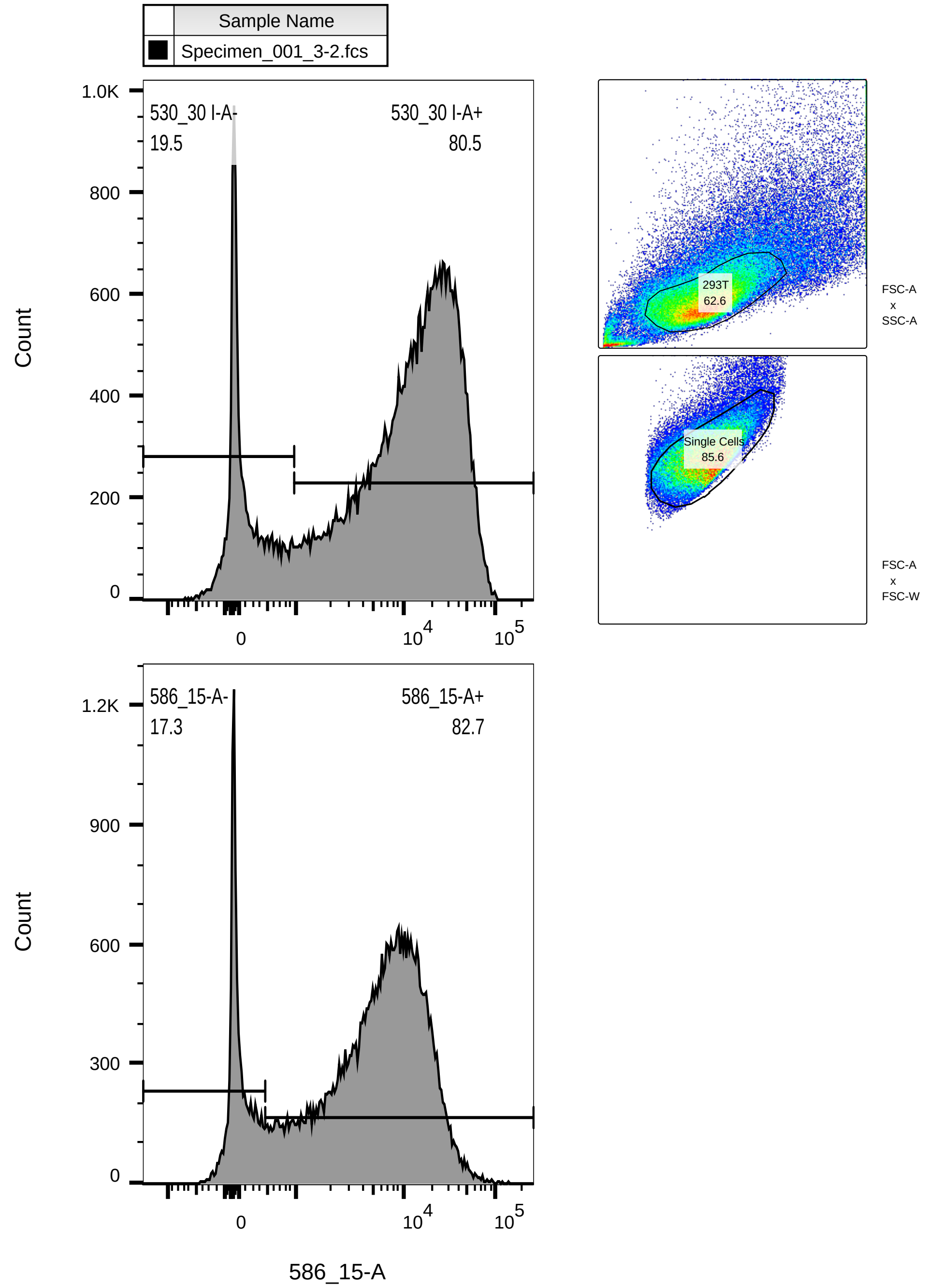
Sample Name
Specimen_001_3-2.fcs
530_30 I-A-
19.5
530_30 I-A+
80.5
1.0K
800
600
400
200
0
Count
0
10^4
10^5
293T
62.6
FSC-A
x
SSC-A
Single Cells
85.6
FSC-A
x
FSC-W
586_15-A-
17.3
586_15-A+
82.7
1.2K
900
600
300
0
Count
0
10^4
10^5
586_15-A

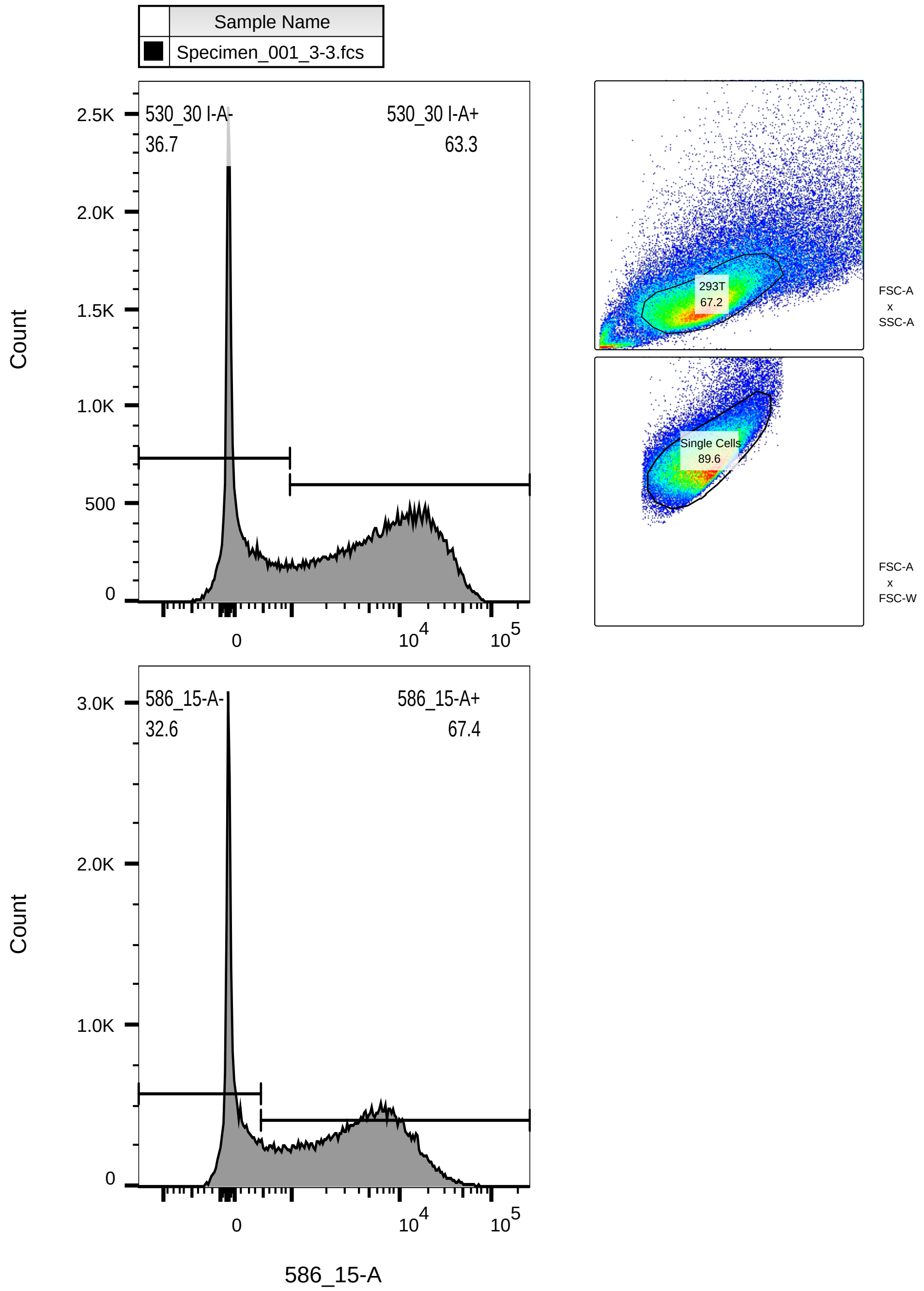
Sample Name
Specimen_001_3-3.fcs
530_30 I-A-
36.7
530_30 I-A+
63.3
2.5K
2.0K
1.5K
1.0K
500
0
Count
0
10^4
10^5
293T
67.2
FSC-A
x
SSC-A
Single Cells
89.6
FSC-A
x
FSC-W
586_15-A-
32.6
586_15-A+
67.4
3.0K
2.0K
1.0K
0
Count
0
10^4
10^5
586_15-A

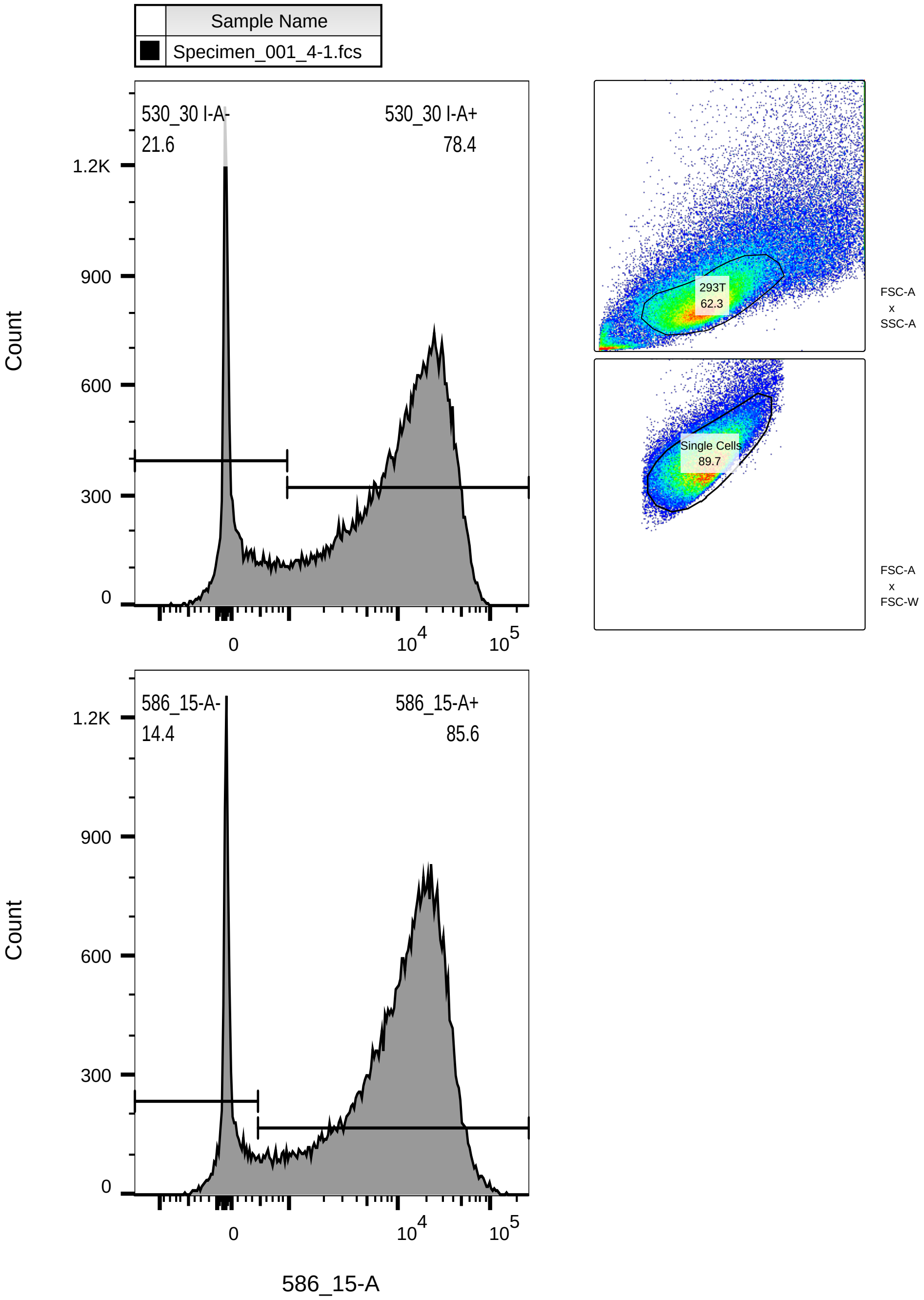
Sample Name
Specimen_001_4-1.fcs
530_30 I-A-
21.6
530_30 I-A+
78.4
Count
1.2K
900
600
300
0
0
10^4
10^5
586_15-A-
14.4
586_15-A+
85.6
586_15-A
293T
62.3
FSC-A
x
SSC-A
Single Cells
89.7
FSC-A
x
FSC-W

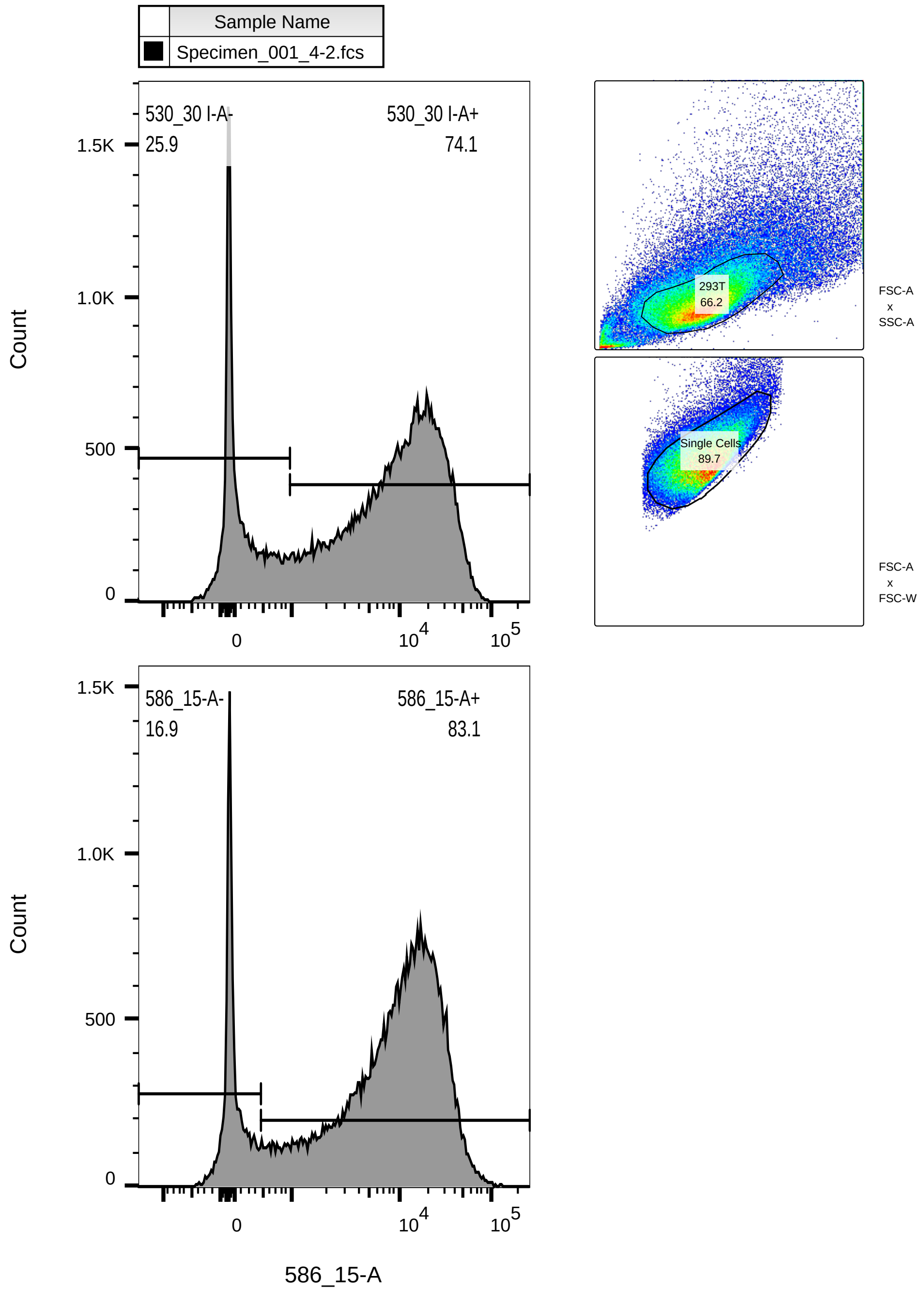
Sample Name
Specimen_001_4-2.fcs
530_30 I-A-
25.9
530_30 I-A+
74.1
Count
1.5K
1.0K
500
0
0
10^4
10^5
293T
66.2
FSC-A x SSC-A
Single Cells
89.7
FSC-A x FSC-W
586_15-A-
16.9
586_15-A+
83.1
586_15-A

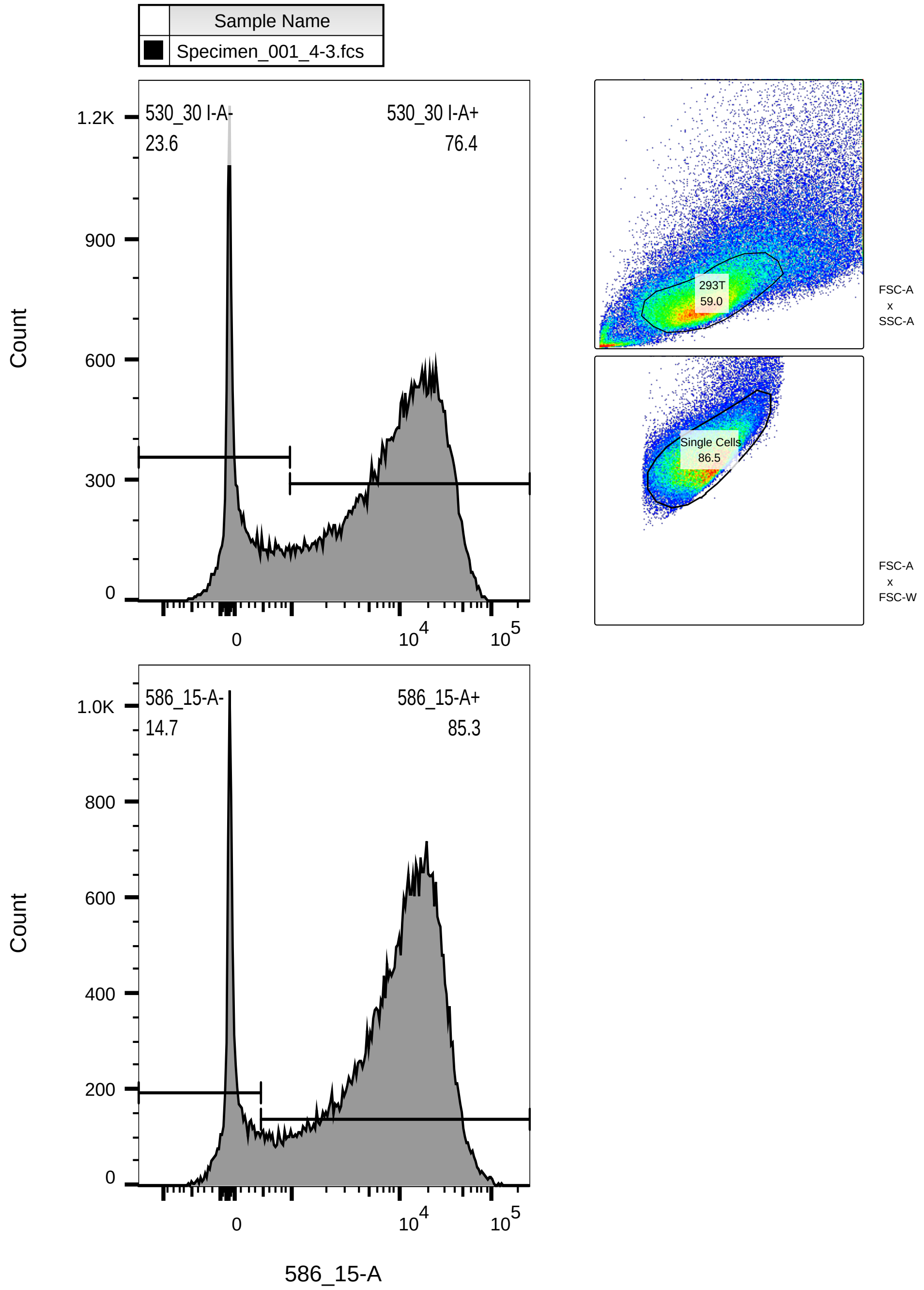

Sample Name
Specimen_001_4-3.fcs
530_30 I-A-
23.6
530_30 I-A+
76.4
1.2K
900
600
300
0
Count
0
10^4
10^5
586_15-A-
14.7
586_15-A+
85.3
1.0K
800
600
400
200
0
Count
0
10^4
10^5
586_15-A
293T
59.0
FSC-A
x
SSC-A
Single Cells
86.5
FSC-A
x
FSC-W

Supplementary Fig. 3 | Gating strategy used for Fig. 2d,e.
