# Supplementary material for: Intron-encoded cistronic transcripts for minimally invasive monitoring of coding and non-coding RNAs
Source: Nat Cell Biol. 2022 Nov 7;24(11):1666–76. doi: 10.1038/s41556-022-00998-6 (PMC9643161; doi:10.1038/s41556-022-00998-6)
Supplement: Supplementary file 2 — Reporting Summary [file 41556_2022_998_MOESM2_ESM.pdf]

nature research

Corresponding author(s): Gil Gregor Westmeyer

Last updated by author(s): Aug 8, 2022

Reporting Summary

Nature Research wishes to improve the reproducibility of the work that we publish. This form provides structure for consistency and transparency in reporting. For further information on Nature Research policies, see Authors & Referees and the Editorial Policy Checklist.

Statistics

For all statistical analyses, confirm that the following items are present in the figure legend, table legend, main text, or Methods section.

n/a	Confirmed	
☐	☒	The exact sample size (*n*) for each experimental group/condition, given as a discrete number and unit of measurement
☐	☒	A statement on whether measurements were taken from distinct samples or whether the same sample was measured repeatedly
☐	☒	The statistical test(s) used AND whether they are one- or two-sided<br>*Only common tests should be described solely by name; describe more complex techniques in the Methods section.*
☒	☐	A description of all covariates tested
☐	☒	A description of any assumptions or corrections, such as tests of normality and adjustment for multiple comparisons
☐	☒	A full description of the statistical parameters including central tendency (e.g. means) or other basic estimates (e.g. regression coefficient) AND variation (e.g. standard deviation) or associated estimates of uncertainty (e.g. confidence intervals)
☐	☒	For null hypothesis testing, the test statistic (e.g. F, t, r) with confidence intervals, effect sizes, degrees of freedom and P value noted<br>*Give P values as exact values whenever suitable.*
☒	☐	For Bayesian analysis, information on the choice of priors and Markov chain Monte Carlo settings
☒	☐	For hierarchical and complex designs, identification of the appropriate level for tests and full reporting of outcomes
☒	☐	Estimates of effect sizes (e.g. Cohen's *d*, Pearson's *r*), indicating how they were calculated

Our web collection on statistics for biologists contains articles on many of the points above.

Software and code

Policy information about availability of computer code

Data collection	Geneious Prime 2019, MikroWin 2000 4.34, BD FACSDiva 6.1.3, QuantStudio 12K Flex
Data analysis	PRISM 9, Geneious Prime 2019, Fiji ImageJ (1.52p), FACSDiva (6.1.3), Image Lab software (v6.1.0 build 7, Bio-Rad), QuantStudio 12K Flex (v1.4), Human Splice Finder (v3.1), NetGene2 (v2.42)

For manuscripts utilizing custom algorithms or software that are central to the research but not yet described in published literature, software must be made available to editors/reviewers. We strongly encourage code deposition in a community repository (e.g. GitHub). See the Nature Research guidelines for submitting code & software for further information.

Data

Policy information about availability of data

All manuscripts must include a data availability statement. This statement should provide the following information, where applicable:

- Accession codes, unique identifiers, or web links for publicly available datasets
- A list of figures that have associated raw data
- A description of any restrictions on data availability

Source data are provided with this paper. All other data supporting the findings of this study are available from the corresponding author on request.

Field-specific reporting

Please select the one below that is the best fit for your research. If you are not sure, read the appropriate sections before making your selection.

☒ Life sciences ☐ Behavioural & social sciences ☐ Ecological, evolutionary & environmental sciences

October 2018

Life sciences study design

All studies must disclose on these points even when the disclosure is negative.

Sample size	Preliminary luciferase-based experiments showed that there was only small variation over independent replicates, so we decided for n>=3.
Data exclusions	Excluded data points were indicated in the raw data tables with a *.
Replication	All experiments were successfully replicated with independent biological samples.
Randomization	This technical report used cell culture as the primary method. Cell culture wells were allocated to the different treatment groups specified by the particular experiment without determining any specific property of the cells in a given well for selecting a specific condition.
Blinding	The experiments were performed, if possible, with master mixes and with multichannel pipettes to exclude unconscious biases during sample preparation. However, the individual human experimenter was not blinded for the conditions as this was not feasible.

Reporting for specific materials, systems and methods

We require information from authors about some types of materials, experimental systems and methods used in many studies. Here, indicate whether each material, system or method listed is relevant to your study. If you are not sure if a list item applies to your research, read the appropriate section before selecting a response.

Materials & experimental systems

n/a	Involved in the study	
☐	☒	Antibodies
☐	☒	Eukaryotic cell lines
☒	☐	Palaeontology
☒	☐	Animals and other organisms
☒	☐	Human research participants
☒	☐	Clinical data

Methods

n/a	Involved in the study	
☒	☐	ChIP-seq
☐	☒	Flow cytometry
☒	☐	MRI-based neuroimaging

Antibodies

Antibodies used	IL-2 Monoclonal Antibody (MQ1-17H12): https://www.thermofisher.com/antibody/product/IL-2-Antibody-clone-MQ1-17H12-Monoclonal/14-7029-81<br>IL-2 Polyclonal Antibody, Biotin: https://www.thermofisher.com/antibody/product/IL-2-Antibody-Polyclonal/13-7028-85
Validation	IL-2 Monoclonal Antibody (MQ1-17H12): Validation shown in 'Advanced verifcation' on https://www.thermofisher.com/antibody/product/IL-2-Antibody-clone-MQ1-17H12-Monoclonal/14-7029-81<br>IL-2 Polyclonal Antibody, Biotin: Validation shown in Bounab, et al. Dynamic single-cell phenotyping of immune cells using the microfluidic platform DropMap. (doi:10.1038/s41596-020-0354-0)

Eukaryotic cell lines

Policy information about cell lines

Cell line source(s)	HEK293T cells (ECACC: 12022001, Sigma-Aldrich), Jurkat E6.1 cells (ECACC 88042803), H9 hESC (WiCELL Research Institute)
Authentication	All cells were purchased from trusted vendors.
Mycoplasma contamination	All cell lines were tested for mycoplasma contamination using MycoAlertTM Mycoplasma Detection Kit (LT07-318, Lonza). In addition, all cell lines were tested every 3 months for contamination by Hoechst 3334, which visualizes extranuclear speckles in case of contamination. All results shown in the manuscript were from cells tested negative for mycoplasma.
Commonly misidentified lines (See ICLAC register)	No commonly misidentified cell lines were used in this study.

Flow Cytometry

Plots

Confirm that:

☒ The axis labels state the marker and fluorochrome used (e.g. CD4-FITC).

☒ The axis scales are clearly visible. Include numbers along axes only for bottom left plot of group (a 'group' is an analysis of identical markers).

☐ All plots are contour plots with outliers or pseudocolor plots.

☒ A numerical value for number of cells or percentage (with statistics) is provided.

Methodology

Sample preparation	Adherent cells from 48-well plates transfection were dissociated with 200 µl Accutase™ and centrifuged at 200 r.c.f. for 5 min at RT, and cell pellets were resuspended in ice-cold 500 µl DPBS containing 0.4% PFA. After an additional washing step with DPBS, the fixated resuspended cells were transferred into conical 5 ml polystyrene round-bottom tubes, including a cell-strainer cap, and were kept on ice.
Instrument	BD FACSaria II (BD Biosciences)
Software	BD FACSDiva Software (Version 6.1.3, BD Biosciences)
Cell population abundance	For FACS analysis, at least 50,000 events were recorded per condition. The smallest fraction of analyzed cells was >5% of total events.
Gating strategy	The main cell population was gated according to their forward scatter and sideward scatter. Afterward, single cells were chosen according to their FSC-A and FSC-W. Subsequently, the transfected cells were gated according to their green fluorescence (530 nm) or red fluorescence (586 nm). These gates were not changed for all subsequent experimental conditions.

☒ Tick this box to confirm that a figure exemplifying the gating strategy is provided in the Supplementary Information.
