# Supplementary figures and images for: Intron-encoded cistronic transcripts for minimally invasive monitoring of coding and non-coding RNAs
Source: Nat Cell Biol. 2022 Nov 7;24(11):1666–76. doi: 10.1038/s41556-022-00998-6 (PMC9643161; doi:10.1038/s41556-022-00998-6)

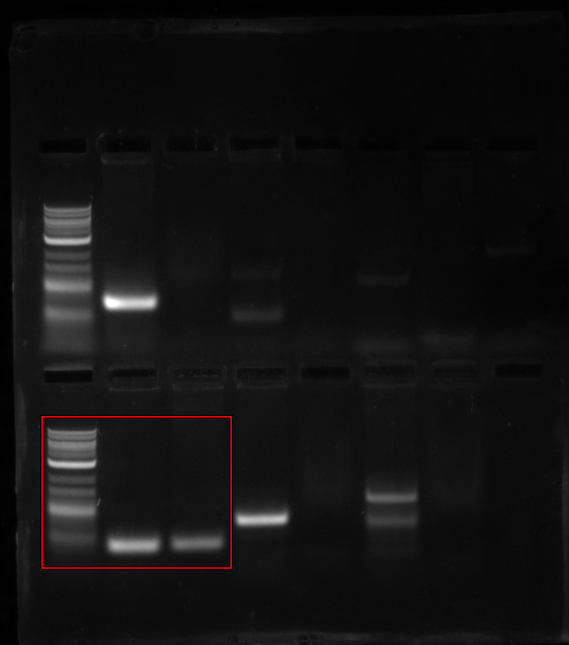

Supplement: Source Data Fig. 4 — Uncropped RT-PCR DNA gels. [file 41556_2022_998_MOESM10_ESM.pdf]

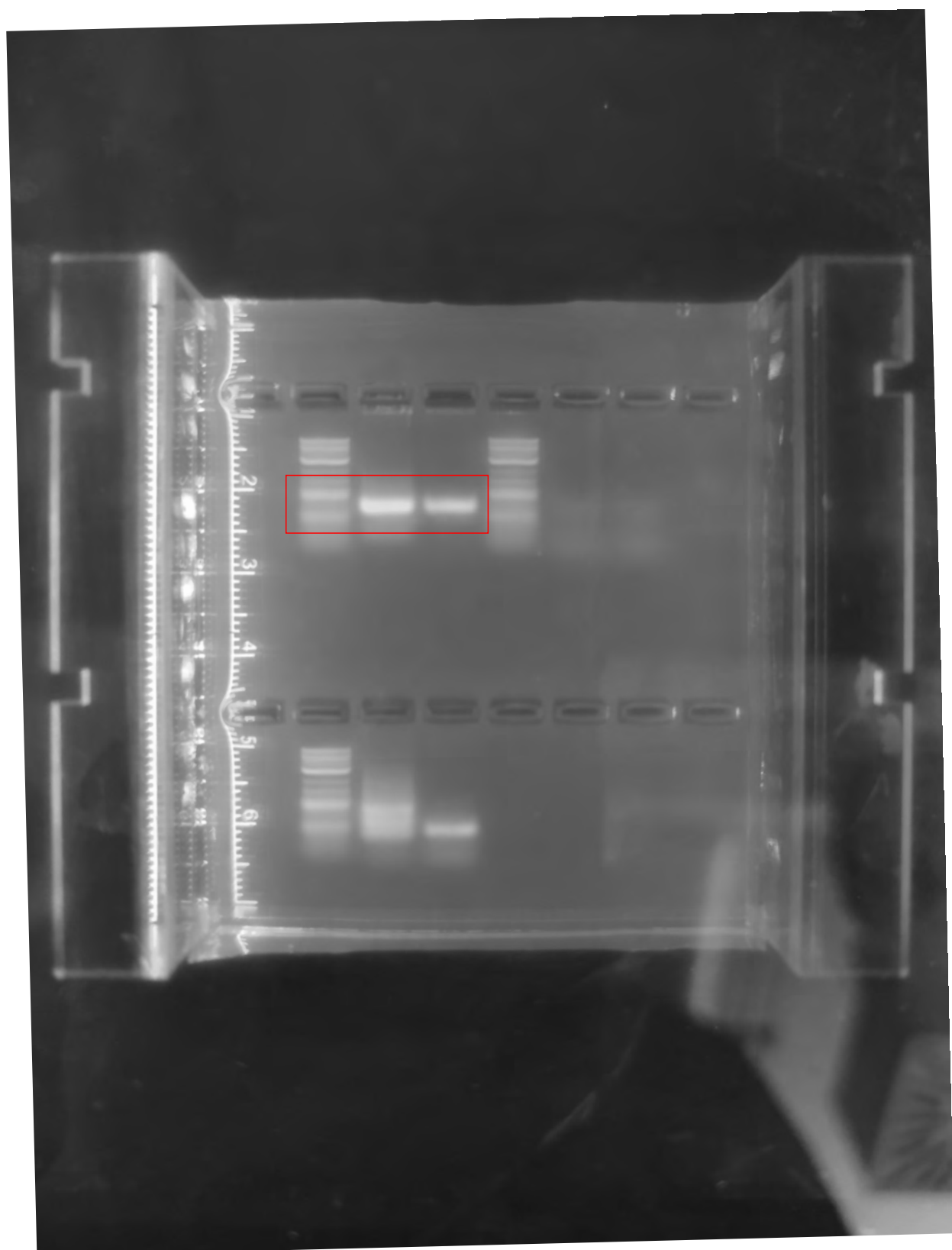

Supplement: Source Data Fig. 5 — Uncropped RT-PCR DNA gels. [file 41556_2022_998_MOESM12_ESM.pdf]

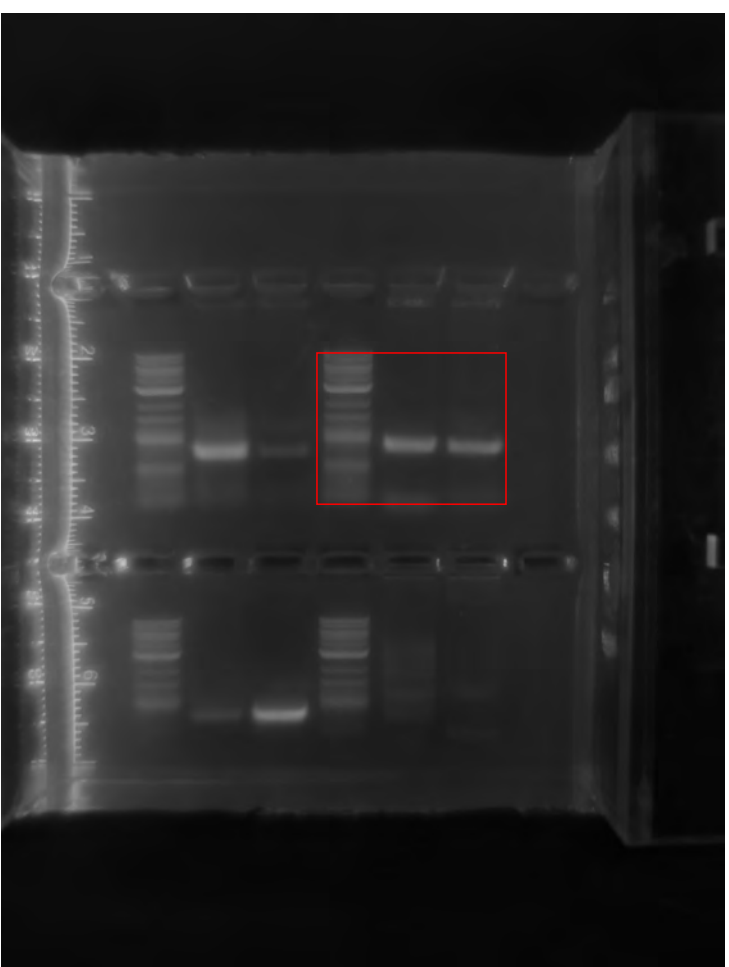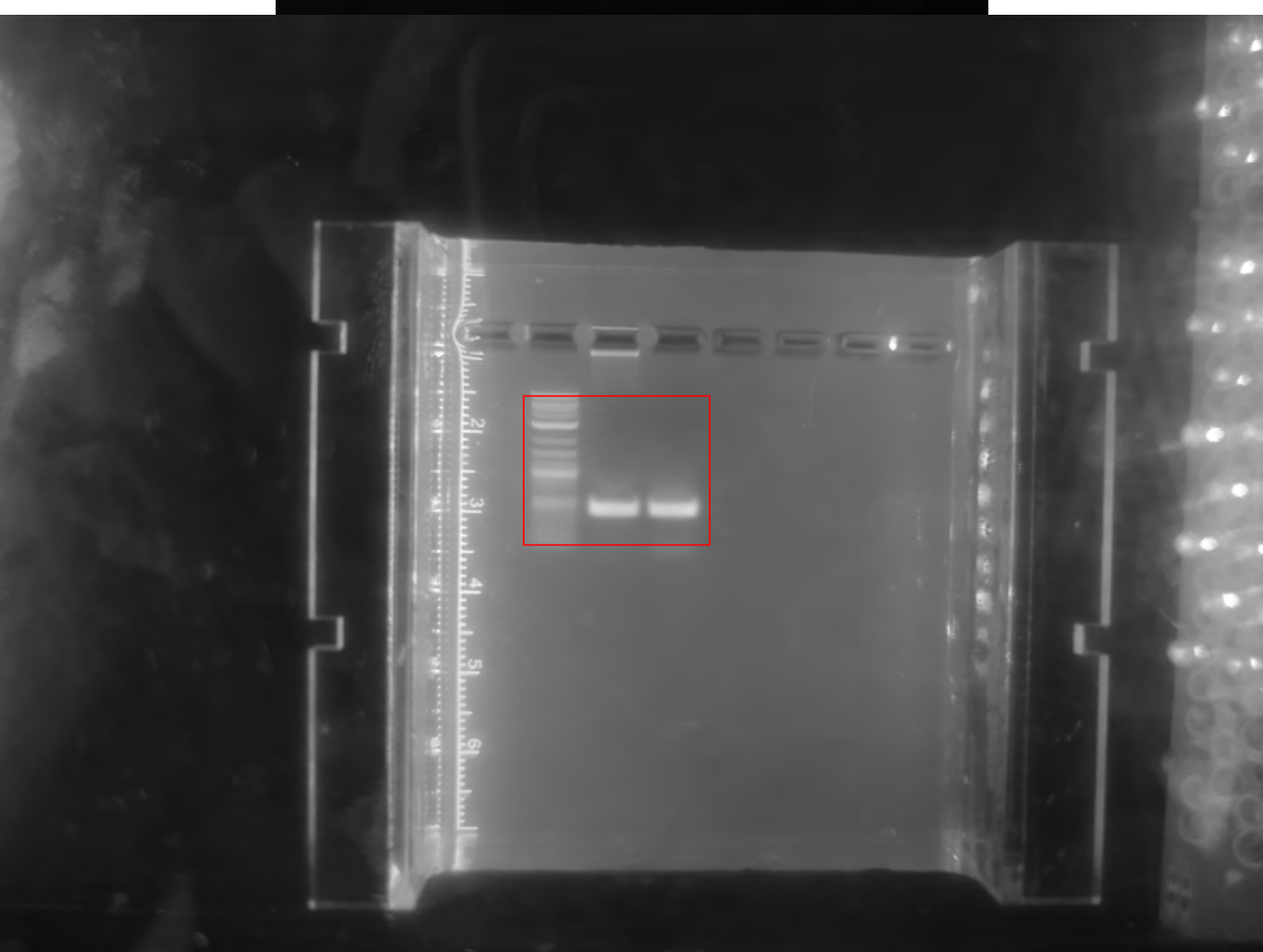

Supplement: Source Data Extended Data Fig. 4 — Uncropped RT-PCR DNA gels. [file 41556_2022_998_MOESM16_ESM.pdf]

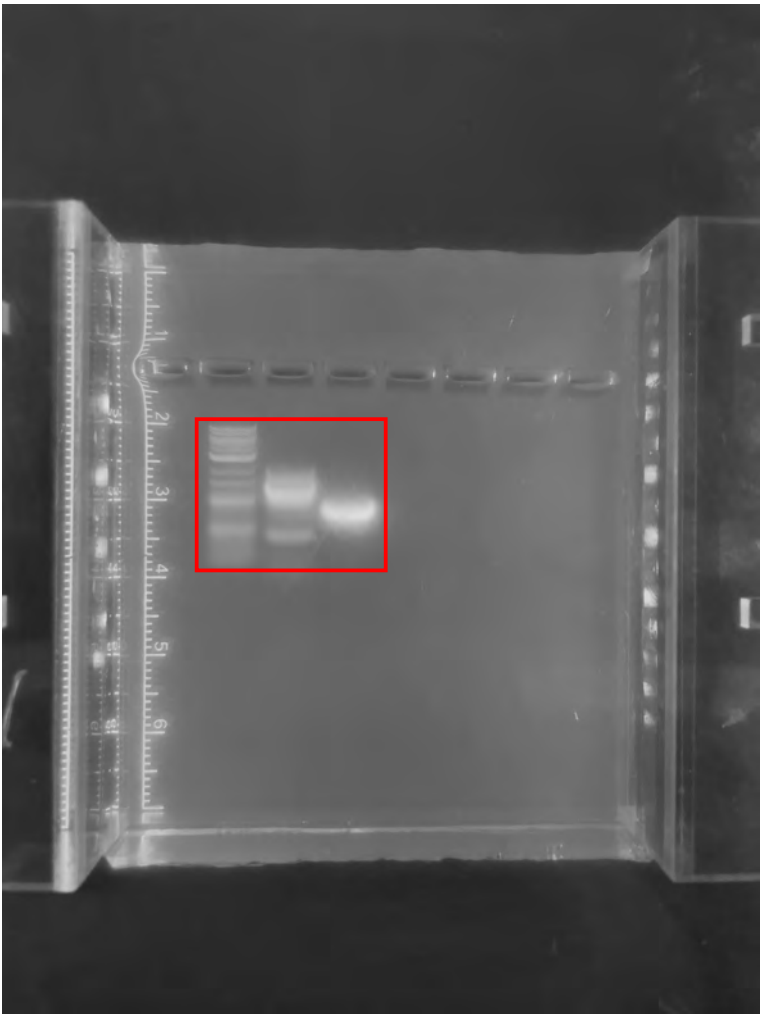

Supplement: Source Data Extended Data Fig. 5 — Uncropped RT-PCR DNA gels. [file 41556_2022_998_MOESM18_ESM.pdf]
